# A combination of pre-infusion serum ferritin, CRP and IL-6 predicts outcome in relapsed/refractory multiple myeloma patients treated with CAR-T cells

**DOI:** 10.3389/fimmu.2023.1169071

**Published:** 2023-04-19

**Authors:** Yang Liu, Xingxing Jie, Li Nian, Ying Wang, Congyue Wang, Jin Ma, Jingjing Jiang, Qingyun Wu, Jianlin Qiao, Wei Chen, Jiang Cao, Zhiling Yan, Ming Shi, Hai Cheng, Feng Zhu, Wei Sang, Depeng Li, Chong Chen, Kailin Xu, Zhenyu Li

**Affiliations:** ^1^ Blood Diseases Institute, Xuzhou Medical University, Xuzhou, Jiangsu, China; ^2^ Department of Hematology, The Affiliated Hospital of Xuzhou Medical University, Xuzhou, Jiangsu, China; ^3^ Jiangsu Key Laboratory of Bone Marrow Stem Cells, Xuzhou, Jiangsu, China; ^4^ Cancer Institute, Xuzhou Medical University, Xuzhou, Jiangsu, China

**Keywords:** chimeric antigen receptor T cell, relapsed/refractory, multiple myeloma, prognostic predictor, inflammation

## Abstract

**Background:**

Chimeric antigen receptor - T (CAR-T) cell therapy has shown remarkable efficacy in patients with relapsed/refractory multiple myeloma (R/R MM). However, a subset of patients still experienced progression or relapse, and the predictors of prognosis are little known. We analyzed the inflammatory markers before CAR-T cell infusion, to clarify their correlation with survival and toxicity.

**Methods:**

This study involved 109 R/R MM patients who received CAR-T therapy between June 2017 and July 2021. Inflammatory markers, including ferritin, c-reactive protein (CRP), and interleukin-6 (IL-6) before CAR-T cell infusion were detected and then categorized by quartiles. Adverse events and clinical outcomes were compared between patients with upper quartile of inflammatory markers and patients with lower three quartiles of inflammatory markers. An inflammatory prognostic index (InPI) based on these three inflammatory markers was developed in this study. Patients were divided into 3 groups according to the InPI score, progression-free survival (PFS) and overall survival (OS) were compared among the groups. In addition, we explored the correlation between cytokine release syndrome (CRS) and pre-infusion inflammatory markers.

**Results:**

We found that the pre-infusion high ferritin (hazard ratio [HR], 3.382; 95% confidence interval [CI], 1.667 to 6.863; *P* = .0007), high CRP (HR, 2.043; 95% CI, 1.019 to 4.097; *P* = .044), and high IL-6 (HR, 3.298; 95% CI, 1.598 to 6.808; *P* = .0013) were significantly associated with inferior OS. The formula of the InPI score was based on the HR value of these 3 variables. Three risk groups were formed: (good, 0 to 0.5 point; intermediate, 1 to 1.5 points; poor, 2 to 2.5 points). Median OS for patients with good, intermediate, and poor InPI was not reached, 24 months, and 4 months, respectively, and median PFS was 19.1 months, 12.3 months, and 2.9 months, respectively. In the cox proportional hazards model, poor InPI remained an independent prognostic factor for PFS and OS. Pre-infusion ferritin was negatively associated with CAR T-cell expansion normalized to baseline tumor burden. Spearman correlation analysis showed that pre-infusion ferritin and IL-6 levels positively correlated with the grade of CRS (*P* = .0369 and *P* = .0117, respectively). The incidence of severe CRS was higher in patients with high IL-6 compared with patients with low IL-6 (26% *vs*. 9%, *P* = .0405). Pre-infusion ferritin, CRP and IL-6 were positively correlated with each peak values within the first month after infusion.

**Conclusions:**

Our results suggest that patients with elevated inflammation markers before CAR-T cell infusion are more likely to have poor prognosis.

## Introduction

Chimeric antigen receptor - T (CAR – T) cells, which could recognize and kill tumor cells through major histocompatibility complex (MHC)-unrestricted pattern, is very promising in the era of immunotherapy (1). Multiple clinical trials have demonstrated unprecedented response rates of anti-BCMA CAR-T therapy in relapsed/refractory multiple myeloma (R/R MM) patients, regardless of previous treatment, ISS stage and cytogenetic risk (2). However, there was significant discrepancy in respect to long-term outcomes, and some patients experienced early progression or relapse (3, 4). Efforts have been made to boost and prolong the efficacy, including *in vitro* enriching memory phenotype T cells through culturing CAR-T cells with PI3K inhibitors (5), combination of γ-secretase inhibitor to increase the BCMA expression on the surface of MM cells (6) and “armed” CAR-T cells to transform an immune-suppressive signal into an immune-stimulatory signal (7).

Although the improvement of CAR-T cells and the exploration of new targets are undoubtedly critical, the identification of prognostic markers is also needed to help us distinguishing patients with poor prognosis for early intervention. It is generally believed that inflammation is critical for the oncogenesis and progression of tumor (8). A peripheral pro-inflammatory status has been reported to be related with worse outcomes in tumor patients treated with immune checkpoint inhibitors (9–11). Inflammatory markers, such as ferritin, c-reactive protein (CRP) and interleukin-6 (IL-6) have been widely proved to be related with cytokine release syndrome (CRS) during CAR-T cell therapy (12–15). The occurrence of severe CRS is associated with high early mortality (16), and the CRS-related complications such as delayed hematopoietic recovery, coagulopathy and cardiac disorders will also dispose patients to poor outcomes (17–19). To date, there are limited data regarding the correlation of circulating inflammatory markers and the prognosis of CAR-T therapy in the setting of MM. Herein, we conducted a retrospective study to clarify their correlation in a relatively large cohort of 109 R/R MM patients.

## Patients and methods

### Study population

This retrospective study included 109 patients with R/R MM treated with anti-BCMA CAR-T cells alone (Chinese Clinical Trial Registry, ChiCTR-1900026219) or combined with anti-CD19 CAR-T cells (ChiCTR-OIC-17011272) at the Affiliated Hospital of Xuzhou Medical University between June 2017 and July 2021. This study was approved by the Ethics Committee of Affiliated Hospital of Xuzhou Medical University and was conducted in accordance with the Declaration of Helsinki. The detailed inclusion and exclusion criteria could refer to previous studies (20, 21). Lymphodepletion conditioning chemotherapy was carried out in all patients, the regimen was fludarabine (30 mg/m^2^/d, days -5 to -3) and cyclophosphamide (750 mg/m^2^/d, day -5).

### Data collection and therapeutic evaluation

Disease characteristics of patients were collected at enrollment, including age, gender, MM type, prior treatment, cytogenetic abnormalities. Laboratory data was obtained by retrieving electronic medical records. Baseline lactate dehydrogenase, albumin and beta-2 microglobulin data were defined as the latest data within 15 days prior to lymphodepletion. Baseline values of ferritin, CRP, and IL-6 were collected within 3 days before the CAR-T cell infusion, peak values were collected during the first month after infusion. CAR-T cell counts in peripheral blood were measured by flow cytometry at day 7, day 14, day 21, and day 28 post infusion. Efficacy was assessed according to the International Myeloma Working Group criteria (22). The severity of cytokine release syndrome (CRS) was evaluated according to the ASTCT consensus (23).

### Statistical analysis

The deadline of follow-up for this study was August 31, 2022. OS was defined as the time from CAR T-cell infusion to death of any cause. Progression-free survival (PFS) was calculated from infusion to disease progression or death. Duration of response (DOR) was defined as the time from first partial response (PR) to progression or death. Quartile analysis was used to define the patients with high ferritin, high CRP and high IL-6, i.e., the upper quartile defined as high value, the lower three quartiles defined as low value. The difference between categorical variables was analyzed by Fisher’s exact test. The correlation between continuous variables was calculated by Spearman’s rank-order test. The log-rank test was used to compare the survival difference between groups. Factors with a P value <.2 or with clinical significance were included in the multivariate cox proportional hazards model. Two-sided P value <.05 was considered statistically significant. Statistical analysis was performed using SPSS 19.0 (IBM Corp., Armonk, NY, USA).

## Results

### Patient characteristics

Baseline characteristics of 109 patients with R/R MM treated with CAR-T cells are summarized in **Table 1**. The median age was 57 years (range, 30 to 70 years). 59% of the patients were male. At enrollment, 32 (29%) of the patients had extramedullary disease (EMD), 22 (20%) had high-risk cytogenetics aberrations, 37 (34%) had revised international staging system (R-ISS) stage III diseases. Patients had a median of 4 lines of prior therapy. A total of 28% patients received prior autologous hematopoietic stem cell transplantation. The pre-infusion median ferritin was 469.2ng/mL (interquartile range [IQR], 251.8 – 882.3 ng/mL), and 62 (57%) patients had ferritin above the upper limit of normal (ULN). Median CRP was 5mg/L (IQR, 1.9 – 20.3 mg/L), and above the ULN in 53 (49%) patients. Median IL-6 was 7.6pg/mL (IQR, 3 – 14.1 pg/mL), and above the ULN in 55 (50%) patients.

**Table 1:** Baseline characteristics of 109 patients.

Variable	Overall (N = 109)
**Age, years, median (range)**	57 (30 - 72)
**Gender, Male, n (%)**	64 (59%)
**Extramedullary disease, n (%)**	32 (29%)
MM type, n (%)	
IgG	47 (43%)
IgA	22 (20%)
IgD	8 (7%)
Light chain	28 (26%)
Nonsecretory	4 (4%)
High-risk cytogenetics*, n (%)	22 (20%)
R-ISS, n (%)	
Stage I + II	72 (66%)
Stage III	37 (34%)
**High tumor burden^†^, n (%)**	27 (25%)
**Prior lines of therapy, median (range)**	4 (1 - 17)
**Prior ASCT, n (%)**	31 (28%)
CAR construct, n (%)	
CD19 + BCMA	66 (61%)
BCMA	43 (39%)
**Pre-infusion ferritin, ng/mL, median (range)**	469.2 (14.6 - 5000)
**Pre-infusion CRP, mg/L, median (range)**	5 (0.2 – 241.7)
**Pre-infusion IL-6, pg/mL, median (range)**	7.6 (1 - 60)
**Pre-LD LDH, U/L, median (range)**	205 (110 - 2101)
**Pre-LD β2-MG, ng/ml, median (range)**	2970 (838 - 20000)
**Pre-LD albumin, g/L, median (range)**	39.3 (22.1 - 60.1)

*High-risk: presence of del(17p) and/or translocation t (4;14) and/or translocation t (14;16).

†High tumor burden: defined as ≥ 50% clonal plasma cells or bone marrow plasma cells.

R-ISS, revised - international staging system; ASCT, autologous hematopoietic stem cell transplantation; CRP, c-reactive protein; IL-6, interleukin-6; Pre-LD, pre - lymphodepletion; LDH, lactate dehydrogenase; β2-MG, beta-2 microglobulin.

### Correlation between pre-infusion inflammatory markers and patient characteristics

Quartiles method was used to classify patients with high ferritin (> 882.3 ng/mL), high CRP (> 20.3 mg/L) and high IL-6 (> 14.1 pg/mL), i.e., the upper quartile was defined as high value. We then analyzed the correlation between these inflammatory markers and patients’ clinical and biological indicators. Both age, gender, high-risk cytogenetic, prior treatment and disease stage had no correlation with high-level pre-infusion inflammatory markers (**Table S1**). Interestingly, a significant higher proportion of patients with light chain myeloma had high ferritin (46% *vs*. 17%, *P* = .0021), high CRP (39% *vs*. 20%, *P* = .039), and high IL-6 (39% *vs*. 20%, *P* = .039) compared with those with non-light chain myeloma (**Table S1**). Patients with high ferritin had a higher tumor burden (median plasma cells in bone marrow, 38% *vs* 11%, *P* = .003) than those with low ferritin, also, there was a weak but significant association between ferritin levels and tumor burden (Spearman r = 0.2543, *P* = .0076) (**Table S1** and **Figure S1**). However, we found no correlation between pre-infusion CRP and IL-6 with tumor burden.

### Relationship between pre-infusion inflammatory markers and treatment response

The overall response rate was 85% (93/109) within 3 months after the infusion of CAR-T cells. Seventy-nine patients achieved a very good partial response (VGPR) or better response, and these patients had lower pre-infusion ferritin and IL-6, but not statistically significant (**Figure S2**). Except for high ferritin tended to be associated with decreased VGPR or better response rate (59% *vs*. 77%, *P* = .087), we found no significant association between other inflammatory markers and response rates (**Table S2**).

**Figure 1:**
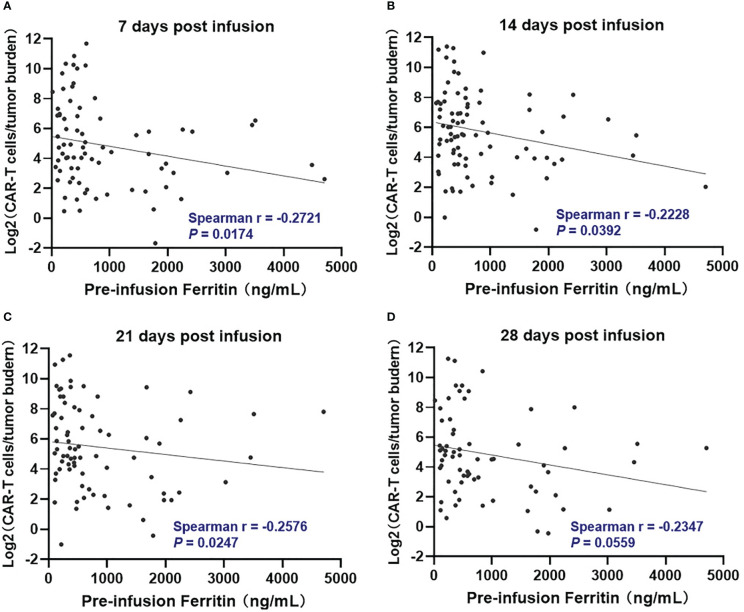
Correlation between pre-infusion ferritin and *in vivo* CAR T-cell expansion normalized to tumor burden during the first month following CAR-T cell infusion. At day 7, 14, and 21 post infusion **(A–C)**, CAR-T cell expansion normalized to baseline tumor burden was negatively correlated with ferritin, and the correlation did not remain at day 28 **(D)**. Tumor burden was defined as percentage of plasma cells in bone marrow. Spearman r value was calculated using the Spearman's correlation test.

We further evaluated if there was a relationship between pre-infusion inflammatory markers and *in vivo* CAR T-cell expansion. However, Pre-infusion ferritin, CRP, and IL-6 were not associated directly with *in vivo* CAR T-cell expansion at indicated time points (days 7, 14, 21, and 28 post infusion) (**Figure S3**). Interestingly, ferritin, but not CRP and IL-6, was significantly (*P* <.05) but modestly (Spearman r < -0.3) associated with lower CAR T-cell expansion normalized to baseline tumor burden at days 7, 14, and 21 post infusion (**Figures 1**, **S4** **).**

### High inflammatory markers were associated with decreased PFS and OS

**Figure 2:**
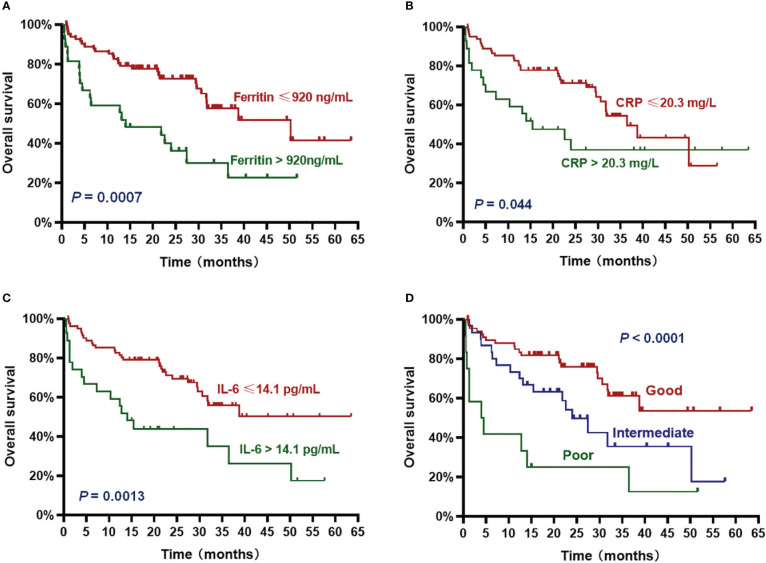
Overall survival (OS) according to inflammatory markers and InPI index. The patients with ferritin > 920 ng/mL **(A)**, CRP > 20.3 mg/L **(B)**, IL-6 > 14.1 pg/mL **(C)** and intermediate to poor InPI **(D)** had inferior OS. Survival curves were drawn according to the Kaplan-Meier method. The log-rank test was used to compare the difference in survival probability between two groups.

**Figure 3:**
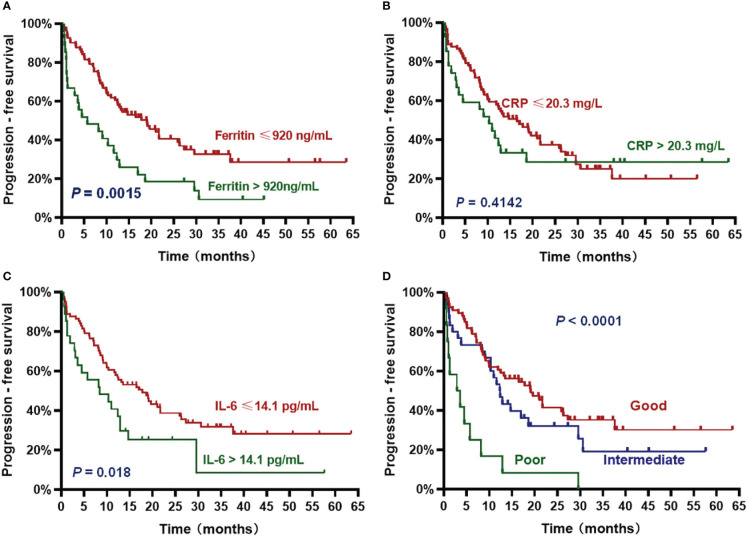
Progression-free survival (PFS) according to inflammatory markers and InPI index. The patients with ferritin > 920 ng/mL **(A)**, IL-6 > 14.1 pg/mL **(C)** and intermediate to poor InPI **(D)** had inferior PFS. No significant association was found between CRP and PFS **(B)**. Survival curves were drawn according to the Kaplan-Meier method. The log-rank test was used to compare the difference in survival probability between two groups.

The patients with high ferritin had significantly poorer OS and PFS compared with those with low ferritin (median OS: 14 months *vs*. 50.2 months, HR 3.382, *P* = .0007; median PFS: 5.7 months *vs*. 19 months, HR 2.611, *P* = .0015) (**Figures 2A**, **3A**). High IL-6 also had similar adverse effects on OS and PFS (median OS: 14 months *vs*. not reached (NR), HR 3.298, *P* = .0013; median PFS: 8.4 months *vs*. 17.8 months, HR 2.026, *P* = .018) (**Figures 2C**, **3C**). Patients with high CRP had inferior OS than patients with low CRP (median of 15.4 months *vs*. 36.5 months, HR 2.043, *P* = .044), but the difference in PFS was not significant (median of 10.3 months *vs*. 16.5 months, HR 1.261, *P* = .4142) (**Figures 2B**, **3B**).

Based on the HR values of ferritin, CRP and IL-6, inflammatory prognostic index (InPI) was developed, 0.5 point was assigned to high CRP, and 1 point was each assigned to high ferritin and high IL-6. According to the InPI score, patients were divided into 3 risk categories: good, 0 to 0.5 point; intermediate, 1 to 1.5 points; poor, 2 to 2.5 points. 67 (61%) of the patients had good InPI, 30 (28%) had intermediate InPI, and 12 (11%) had poor InPI. The median OS for patients with good, intermediate, and poor InPI was NR, 24 months (95% CI, 17.3 months to 30.7 months), and 4 months (95% CI, 0 to 9.4 months), respectively, and median PFS was 19.1 months (95% CI, 12.2 months to 26.0 months), 12.3 months (95% CI, 9.8 months to 14.9 months), and 2.9 months (95% CI, 0 to 6.8 months), respectively (both *P* <.0001) (**Figures 2D**, **3D**).

To further determine whether InPI index was an independent prognostic factor for PFS and OS, we introduced potential influence covariates, including age, gender, EMD, number of therapy lines, type of MM, high-risk cytogenetic, R-ISS stage, tumor burden and InPI into cox proportional hazard model. The results showed that high InPI score still had independent adverse influence on OS (*P* = .009) and PFS (*P* = .01) even after adjusting for tumor burden (**Table 2**). In addition, high tumor burden was independent risk factor for PFS (HR: 2.512, 95% CI: 1.408 – 4.48, *P* = .002) and OS (HR: 2.249, 95% CI: 1.091 – 4.637, *P* = .028). EMD was independent risk factor for OS (HR: 2.077, 95% CI: 1.015 – 4.251, *P* = .046).

**Table 2:** Univariate and multivariate analysis of PFS and OS.

	Progression-free survival (PFS)				Overall survival (OS)			
	Univariate		Multivariate		Univariate		Multivariate	
	HR (95% CI)	P	HR (95% CI)	P	HR (95% CI)	P	HR (95% CI)	P
**Age, >60 years**	0.888 (0.548 – 1.44)	0.6301			0.9689 (0.519 – 1.808)	0.9209		
**Gender, male**	1.117 (0.699 – 1.783)	0.644			1.059 (0.590 – 1.901)	0.8482		
**Prior lines of therapy, ≥ 6**	1.359 (0.802 – 2.305)	0.2541			1.317 (0.687 – 2.524)	0.4069		
**MM type, Light chain**	1.456 (0.839 – 2.528)	0.182			2.165 (1.075- 4.36)	0.0305	1.909 (0.915 – 3.983)	0.085
**Extramedullary disease**	1.788 (1.03 – 3.103)	0.039			2.727 (1.347 - 5.519)	0.0053	2.077 (1.015 – 4.251)	0.046
**Cytogenetics, high-risk**	1.67 (0.88 – 3.168)	0.1167			1.614 (0.749 – 3.48)	0.2218		
**High tumor burden**	3.456 (1.867 – 6.399)	< 0.0001	2.512 (1.408 – 4.48)	0.002	2.68 (1.315 – 5.463)	0.0067	2.249 (1.091 – 4.637)	0.028
**Pre-LD LDH, > ULN**	1.252 (0.7771 – 2.018)	0.3555			0.9592 (0.5322 – 1.729)	0.8896		
**Pre-LD β2-MG, ≥ 5500ng/ml**	1.295 (0.7013 – 2.393)	0.4083			2.386 (1.121 – 5.078)	0.024		
**Pre-LD serum albumin, < 35 g/L**	1.43 (0.847 – 2.415)	0.1806			1.39 (0.7329 – 2.637)	0.3132		
**R-ISS, stage III**	1.159 (0.709 – 1.893)	0.5562			1.266 (0.691 – 2.318)	0.4459		
**InPI**		<0.0001		0.009		<0.0001		0.01
**good**	Ref.		Ref.		Ref.		Ref.	
**intermediate**	1.367 (0.813 – 2.298)	0.238	1.095 (0.608 – 1.971)	0.762	2.102 (1.106 – 3.996)	0.023	1.412 (0.672 – 2.967)	0.362
**poor**	4.199 (2.177 – 8.102)	<0.0001	3.689 (1.571 – 8.664)	0.003	4.957 (2.316 – 10.61)	<0.0001	4.85 (1.736 – 13.553)	0.003

InPI, inflammatory prognostic index; ULN, upper limit of normal.

### High inflammatory markers were associated with decreased DOR

Pre-infusion increases in ferritin and IL-6, but not CRP, were significantly associated with decreased DOR (HR 2.269, 95% CI 1.152 to 4.469, *P* = .0179, for high ferritin; HR 2.224, 95% CI 1.130 to 4.377, *P* = .0207, for high IL-6) (**Figures S5A–C**). The median DOR for patients with good, intermediate, and poor InPI was 21.1 months (95% CI, 14.1 months to 28.1 months), 13.8 months (95% CI, 6.4 months to 21.2 months), and 4 months (95% CI, 1.2 months to 6.8 months), respectively (**Figure S5D**).

### The correlation between pre-infusion inflammatory markers and CRS

**Figure 4:**
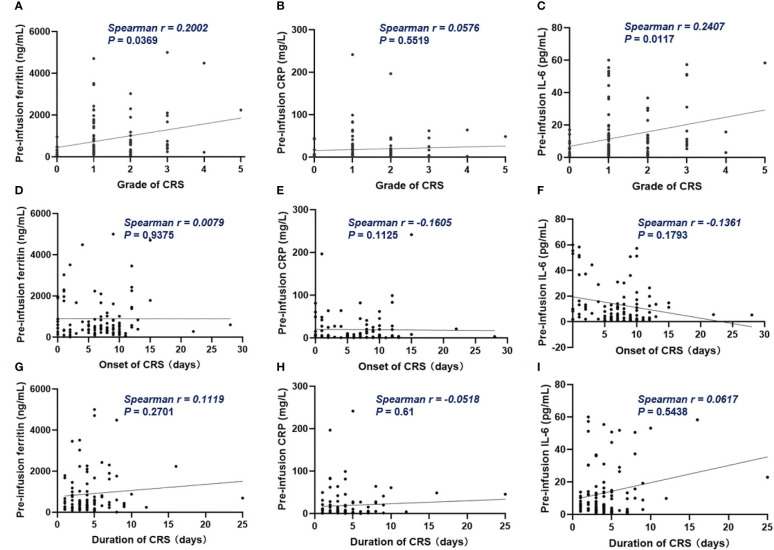
The correlation between inflammatory markers and CRS. **(A, C)** Pre-infusion ferritin and IL-6 correlated with the grade of CRS. **(B)** Pre-infusion CRP had no correlation with the grade of CRS. **(D–I)** The pre-infusion ferritin, CRP and IL-6 had no correlation with the onset and the duration of CRS. Spearman r value was calculated using the Spearman’s correlation test.

**Figure 5:**
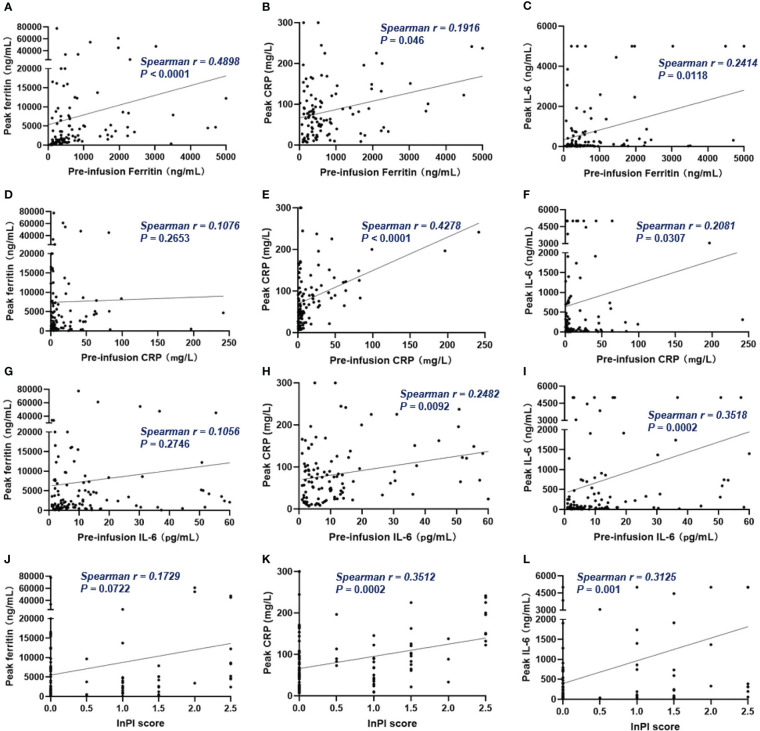
The correlation between pre-infusion inflammatory markers, InPI score and the post-infusion peak inflammatory markers. **(A–C)** Pre-infusion levels of ferritin correlated with the peak values of ferritin, CRP and IL-6. **(D, G)** Pre-infusion CRP and IL-6 had no correlation with peak ferritin. **(E, F)** Pre-infusion CRP correlated with peak CRP and IL-6. **(H, I)** Pre-infusion IL-6 correlated with peak CRP and IL-6. **(J–L)** InPI score positively correlated with peak ferritin, CRP, and IL-6. Spearman r value was calculated using the Spearman’s correlation test.

Of all patients, 91% experienced CRS. Grade 3 or higher CRS, defined as severe CRS, occurred in 14 (13%) patients. Median time to onset of CRS was 7d (0 – 28d), median duration of CRS was 4d (1d - 25d). Patients with lower pre-infusion concentration of ferritin and IL-6 were more likely to develop non-severe CRS than severe CRS (**Table 3**). When considering the severity of CRS as continuous variable, the levels of pre-infusion serum IL-6 (Spearman r = 0.241, *P* = .0117) and ferritin (Spearman r = 0.2, *P* = .0369) were positively correlated with the grade of CRS, no correlation was found between CRP concentration and CRS grade (**Figures 4A–C**). In addition, there was no correlation between inflammatory markers and the onset time of CRS or duration of CRS (**Figures 4D–I**). Furthermore, we found that the levels of pre-infusion ferritin, CRP and IL-6 were positively correlated with the post-infusion peak values of each of these markers (Spearman r = 0.49, *P* <.0001; Spearman r = 0.428, *P* <.0001; Spearman r = 0.352, *P* = .0002; respectively) (**Figures 5A, E, I**). Pre-infusion ferritin correlated with the peak values of CRP and IL-6 (**Figures 5B, C**), but the peak ferritin did not correlate with pre-infusion CRPand IL-6 (**Figures 5D, G**). There were positive correlations between IL-6 and CRP, regardless of baseline and peak values (**Figures 5F, H**). The InPI score also had positive correlation with the post-infusion peak levels of ferritin, CRP and IL-6 (**Figures 5J–L**).

**Table 3:** Association between pre-infusion inflammatory markers and CRS.

	No-severe CRS	Severe CRS	P
**Ferritin**			0.1063
> 920 ng/mL (n = 27)	21 (78%)	6 (22%)	
≤ 920 ng/mL (n = 82)	74 (90%)	8 (10%)	
**C-reactive protein**			0.329
> 20.3 mg/L (n = 27)	22 (81%)	5 (19%)	
≤ 20.3 mg/L (n = 82)	73 (89%)	9 (11%)	
**Interleukin-6**			0.0405
> 14.1 pg/mL (n = 27)	20 (74%)	7 (26%)	
≤ 14.1 pg/mL (n = 82)	75 (91%)	7 (9%)	

Severe CRS defined as grade 3 or higher CRS.

Two-sided P values were calculated using the Fisher’s exact test.

## Discussion

Due to the high financial cost and the potentially life-threatening toxicities, it is of particular importance to early identify patients who will not benefit or less benefit from CAR-T cell therapy. Except for EMD, there are still no validated biomarkers for predicting prognosis in R/R MM patients following CAR-T therapy.

A cohort of 17 patients treated with LCAR-B38M revealed that EMD and anti-CAR T antibody constituted risk factors for early recurrence and progression (24). Que and colleagues found that the patients who had more than 6 lines of prior therapy had shorter PFS and OS, but when incorporating EMD into multivariate analysis, > 6 lines of therapy lost its predictive value, only EMD being an independently significant prognostic factor (25). Recently, zhang et al. reported that EMD, light chain type, high-risk cytogenetics, and > 3 prior therapeutic lines were independent risk factors of PFS, ECOG score of 2 and light chain type were independent risk factors of OS (26). Our results also showed that EMD was an independent risk factor that associated with OS. Besides, light chain type MM was identified as one of the risk factors for inferior OS in the univariate analysis, a marginal significant correlation remained in the multivariate analysis, though the exact mechanism is not clear. We think it deserve further study to verify and make clear of these findings.

An in-depth analysis from ZUMA-1 study in large B-cell lymphoma demonstrated that the levels of ferritin and IL-6 before CAR-T cell infusion had negative correlation with durable remission rates (27). One real world study of Axicabtagene Ciloleucel in patients with R/R B-cell non-Hodgkin lymphoma suggested that low CRP levels at baseline was associated with better response (28). However, the effect of inflammation on the prognosis of CAR-T therapy has not been reported in myeloma patients. Hence, we conducted a *post-hoc* analysis to elucidate their correlation. Our study proved that both pre-infusion high ferritin, high IL-6, and high CRP were risk factors for long-term survival, and patients with high ferritin and high IL-6 had shorter duration of remission. Further, we established a scoring system based on these three inflammatory indicators, defined as InPI index in this context. The InPI index helped us distinguished three groups of patients with different prognosis, the patients with a score of 2 to 2.5 points had the worst survival. Since high inflammation might partly be a proxy for disease burden or aggressive disease, we therefore brought variables that pertain to disease burden into multivariate analysis to determine the independent effect of InPI index. The results showed that after account for high tumor burden and EMD, poor InPI remained an independent predictor for durable remission and survival.

Inflammation has been extensively studied in the malignant progression of tumors, either by acting on cancerous cells or by acting on anti-tumor immunity (29, 30). The pro-inflammatory cytokine IL-6 might impair anti-tumor immunity through multi-aspect. It could restrict the differentiation of Th1 cells and decrease the production of interferon-γ, resulting in less mounting of CD8+ cytotoxic T cells in anti-tumor response (31). Besides, IL-6 could promote the differentiation of Th2 and Th17 cells, thereby tilt anti-tumor immune response to an immunosuppressive response (32, 33). Acute phase proteins such as CRP, fibrinogen and ferritin could be rapidly synthesized in liver after the stimulation of IL-6 (34). Our study also showed that high-level ferritin at baseline might adversely influence *in vivo* CAR-T amplification. These and our results led to a hypothesis that inflammation might be one of the most relevant factors for disease progression and long-term survival in CAR-T therapy, partially by affecting the activation and expansion of effector T cells.

Despite the superior efficacy of CAR-T therapy, its benefits might be offset by the serious adverse effects after infusion. CRS and immune effector cell-associated neurotoxicity syndrome (ICANS) are common adverse effects which are associated with endothelial activation injury and cytokine release. Due to the low incidence of ICANS in our center, we did not further discuss it. According to our recently published data, the occurrence of severe CRS was associated with poorer survival (15). Therefore, it is necessary to identify patients who will develop severe CRS preemptively. In this study, we found that the patients with high ferritin and IL-6 before CAR-T infusion had increased rates of severe CRS, and these inflammatory markers’ baseline levels positively correlated with the peak values after infusion. Peak levels of inflammatory markers had been reported to be correlated with the occurrence and the severity of CRS (35–38). Hay and colleagues reported that the pre-existing endothelial activation before conditioning and CAR-T cell infusion might increase the risk of severe CRS in patients receiving anti-CD19 CAR-T treatment (39). Researchers from University of Pennsylvania found that blood vessel endothelial cells are a key source of IL-6 during CRS (40). Together, these findings indicated that elevated pre-infusion inflammation might increase the risk of developing severe CRS. Although the small sample size of severe CRS in our cohort may limit the statistical power to detect the effect of pre-infusion inflammation on CRS, these might be important markers of concern. As the current predictive power of peak inflammatory markers usually occurs after severe symptoms have already appeared.

To our knowledge, this is the first study to illustrate the importance of pre-infusion inflammation on prognosis in R/R MM patients receiving CAR-T cell therapy. However, there are some limitations to our study. It is a single-center retrospective study. We had limited data to interpret the effect of inflammation at the time of apheresis on prognosis. In addition, because our findings were from a retrospective study, we did not take special interventions to ameliorate inflammation before CAR-T cell infusion. We also appreciate that there will be prospective studies to validate our findings by using commercial CAR-T products.

## Conclusion

In conclusion, pre-infusion inflammation markers were useful predictors of durable remission and long-term survival, and might be risk factors for the subsequent development of severe CRS. Our data suggest that treating patients’ pre-infusion inflammation earlier in their course may improve durability of response to CAR-T cell therapy.

## Data availability statement

The raw data supporting the conclusions of this article will be made available by the authors, without undue reservation.

## Ethics statement

The studies involving human participants were reviewed and approved by Ethics committee of the Affiliated Hospital of Xuzhou Medical University. The patients/participants provided their written informed consent to participate in this study.

## Author contributions

ZL, KX and CC designed the study. YW, WC, JC, ZY, HC, FZ, WS, QW, CC and DL were responsible for patient enrollment. YL, XJ, LN, CW, JM and JJ collected the data. MS was responsible for the generation of CAR-T cells. YL and LN performed statistical analysis. YL wrote the manuscript. ZL, JQ, CC and KX revised the manuscript. All authors contributed to the article and approved the submitted version.
