# Supplementary material for: A combination of pre-infusion serum ferritin, CRP and IL-6 predicts outcome in relapsed/refractory multiple myeloma patients treated with CAR-T cells
Source: Front Immunol. 2023 Apr 19;14:1169071. doi: 10.3389/fimmu.2023.1169071 (PMC10154462; doi:10.3389/fimmu.2023.1169071)
Supplement: Supplementary file 1 [file DataSheet_1.docx]

**A combination of pre-infusion serum ferritin, CRP and IL-6 predicts outcome in relapsed/refractory multiple myeloma patients treated with CAR-T cells**

Supplemental Data

**Table S1.** **Patients’ clinical and biological characteristics according to pre-infusion inflammatory markers.**

|  | **Ferritin** | | |  | | | **CRP** | | |  | | | **IL-6** | | |
| --- | --- | --- | --- | --- | --- | --- | --- | --- | --- | --- | --- | --- | --- | --- | --- |
|  | **High（n=27）** | **Low（n=82）** | ***P*** | |  | **High**  **（n=27）** | | **Low（n=82）** | ***P*** | |  | **High （n=27）** | | **Low （n=82）** | ***P*** |
| **Age, years, median (range)** | 57 (30-66) | 57 (31-72) | 0.75 | |  | 57 (30-72) | | 57 (31-70) | 0.747 | |  | 57 (30-72) | | 57 (31-70) | 0.641 |
| **Gender, Male, n (%)** | 14 (52%) | 50 (61%) | 0.5 | |  | 15 (56%) | | 49 (60%) | 0.822 | |  | 15 (56%) | | 49 (60%) | 0.822 |
| **Prior lines of therapy, median (range)** | 5 (2-11) | 4 (1 - 17) | 0.059 | |  | 4 (2-17) | | 4 (1 - 17) | 0.780 | |  | 4 (2-17) | | 4 (1 - 17) | 0.978 |
| **Prior ASCT, n (%)** | 10 (37%) | 21 (26%) | 0.326 | |  | 11 (41%) | | 20 (24%) | 0.102 | |  | 9 (33%) | | 22 (27%) | 0.624 |
| **Plasma cells in bone marrow (%)** | 38 (1-86.5) | 11 (0.5-93) | 0.003 | |  | 20 (0.8-79) | | 16 (0.5-93) | 0.695 | |  | 16 (0.8-79) | | 16 (0.5-93) | 0.441 |
| **MM type, n (%)** |  |  | 0.002 | |  |  | |  | 0.039 | |  |  | |  | 0.039 |
| **Non-Light chain** | 14 (52%) | 67 (82%) |  | |  | 16 (59%) | | 65 (79%) |  | |  | 16 (59%) | | 65 (79%) |  |
| **Light chain** | 13 (48%) | 15 (18%) |  | |  | 11 (41%) | | 17 (21%) |  | |  | 11 (41%) | | 17 (21%) |  |
| **R-ISS stage Ⅲ** | 13 (48%) | 24 (29%) | 0.1 | |  | 12 (44%) | | 25 (30%) | 0.242 | |  | 13 (48%) | | 24 (29%) | 0.1 |
| **Extramedullary disease, n (%)** | 9 (33%) | 23 (28%) | 0.631 | |  | 11 (41%) | | 21 (26%) | 0.150 | |  | 10 (37%) | | 22 (27%) | 0.337 |
| **High-risk cytogenetics*, n (%)** | 6 (22%) | 16 (20%) | 0.785 | |  | 3 (11%) | | 19 (23%) | 0.269 | |  | 5 (19%) | | 17 (21%) | 1.0 |

* High-risk: presence of del(17p) and/or translocation t (4;14) and/or translocation t (14;16).

| **Response category** | **Very good partial response (VGPR) or better** | | ***P*** | **Complete response (CR) or better** | | ***P*** |
| --- | --- | --- | --- | --- | --- | --- |
|  | **≥ VGPR** | **< VGPR** |  | **≥ CR** | **< CR** |  |
| **Ferritin** |  |  | 0.0870 |  |  | 0.2677 |
| > 920 ng/mL (n = 27) | 16 (59%) | 11 (41%) |  | 13 (48%) | 14 (52%) |  |
| ≤ 920 ng/mL (n = 82) | 63 (77%) | 19(23%) |  | 50 (61%) | 32 (39%) |  |
| **C-reactive protein** |  |  | 0.4625 |  |  | 0.5064 |
| > 20.3 mg/L (n = 27) | 18 (67%) | 9 (33%) |  | 14 (52%) | 13 (48%) |  |
| ≤ 20.3 mg/L (n = 82) | 61 (74%) | 21 (26%) |  | 49 (60%) | 33 (40%) |  |
| **Interleukin-6** |  |  | 0.2209 |  |  | 0.2677 |
| > 14.1 pg/mL (n = 27) | 17 (63%) | 10 (37%) |  | 13 (48%) | 14 (52%) |  |
| ≤ 14.1 pg/mL (n = 82) | 62 (76%) | 20 (24%) |  | 50 (61%) | 32 (39%) |  |

**Table S2. Association between inflammation markers and treatment responses.**

Two-sided *P* values were calculated using the Fisher’s exact test


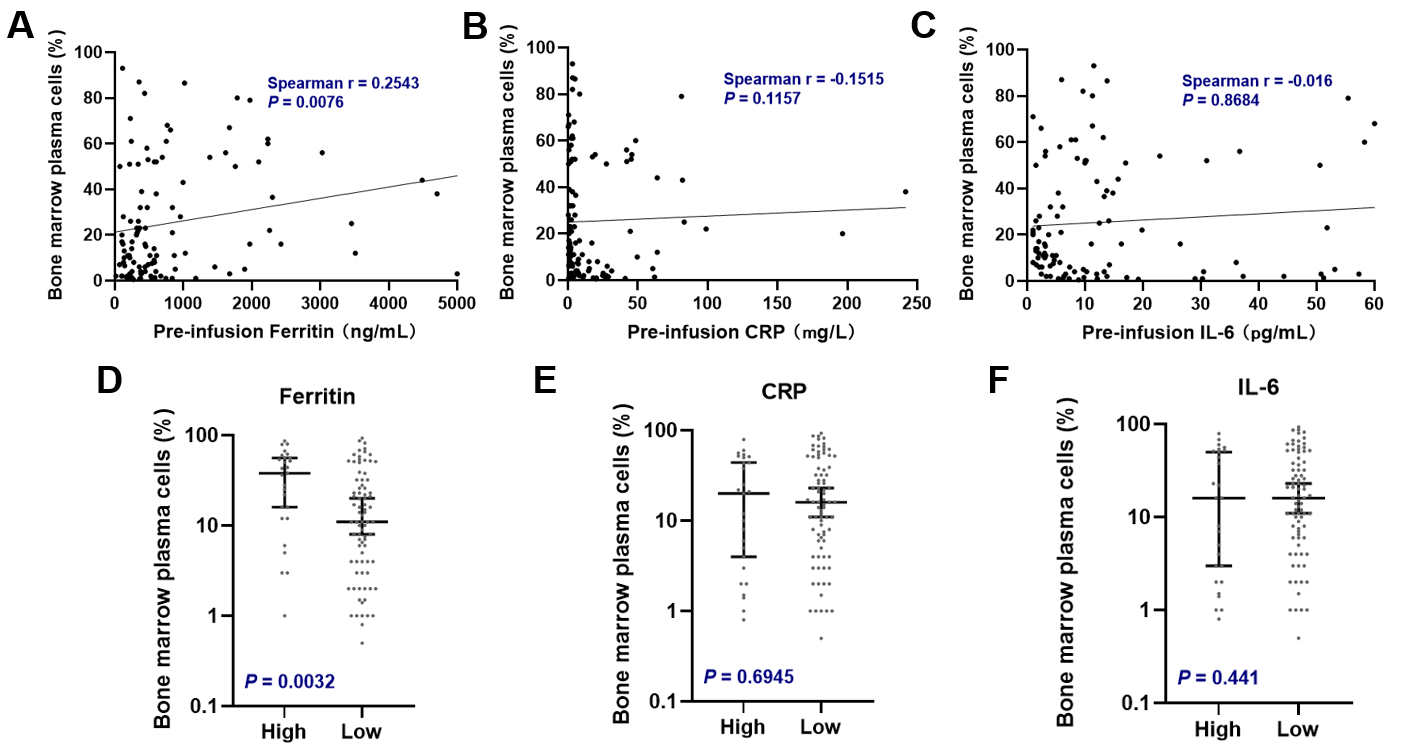
 **Figure. S1. Correlation between pre-infusion inflammatory markers and tumor burden.** (A-C). Spearman r value was calculated using the Spearman's correlation test. (D-F). Two-sided *P* values were calculated using the Mann–Whitney U test


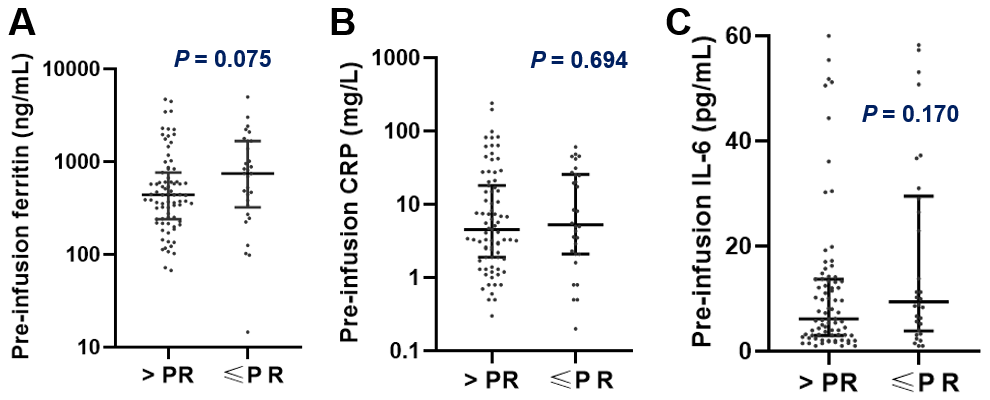


**Figure. S2. Pre-infusion inflammatory markers in different response groups.** Two-sided *P* values were calculated using the Mann–Whitney U test.


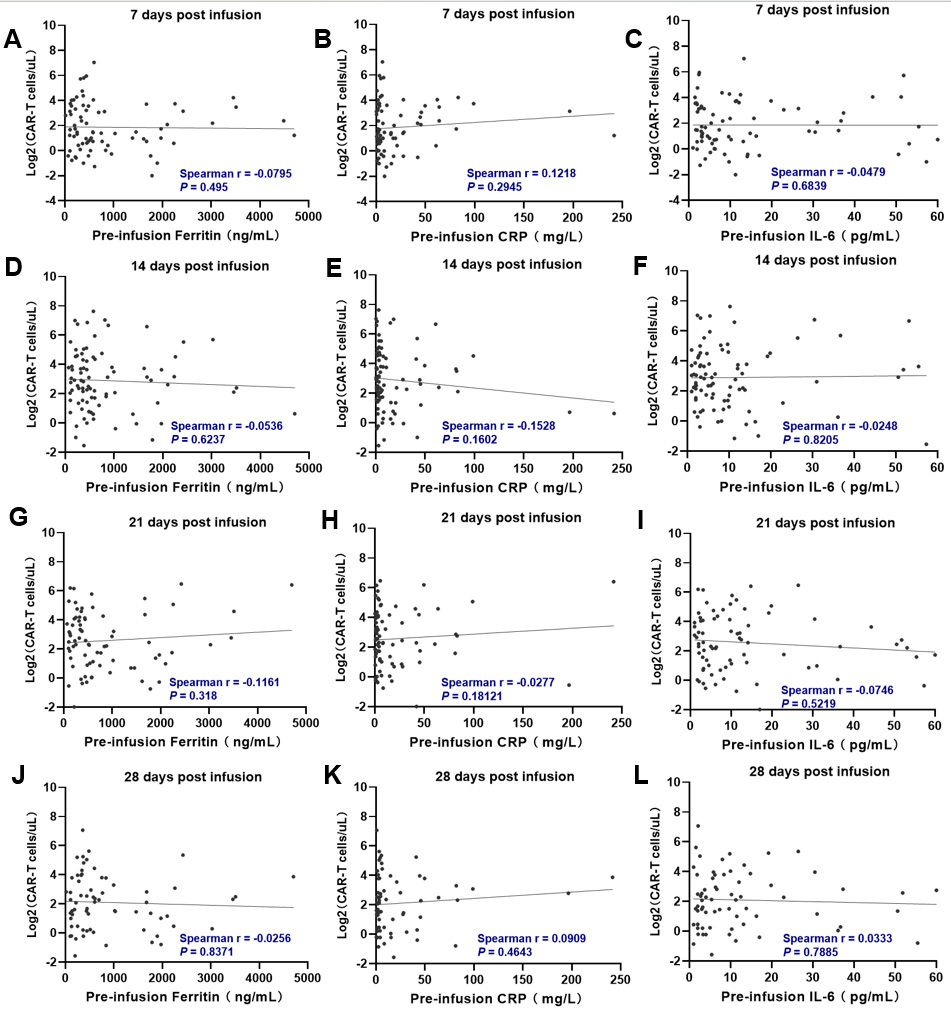
 **Figure S3: Correlation between the pre-infusion inflammatory markers and in vivo CAR T-cell expansion during the first month following CAR-T cell infusion.** Spearman r value was calculated using the Spearman's correlation test.


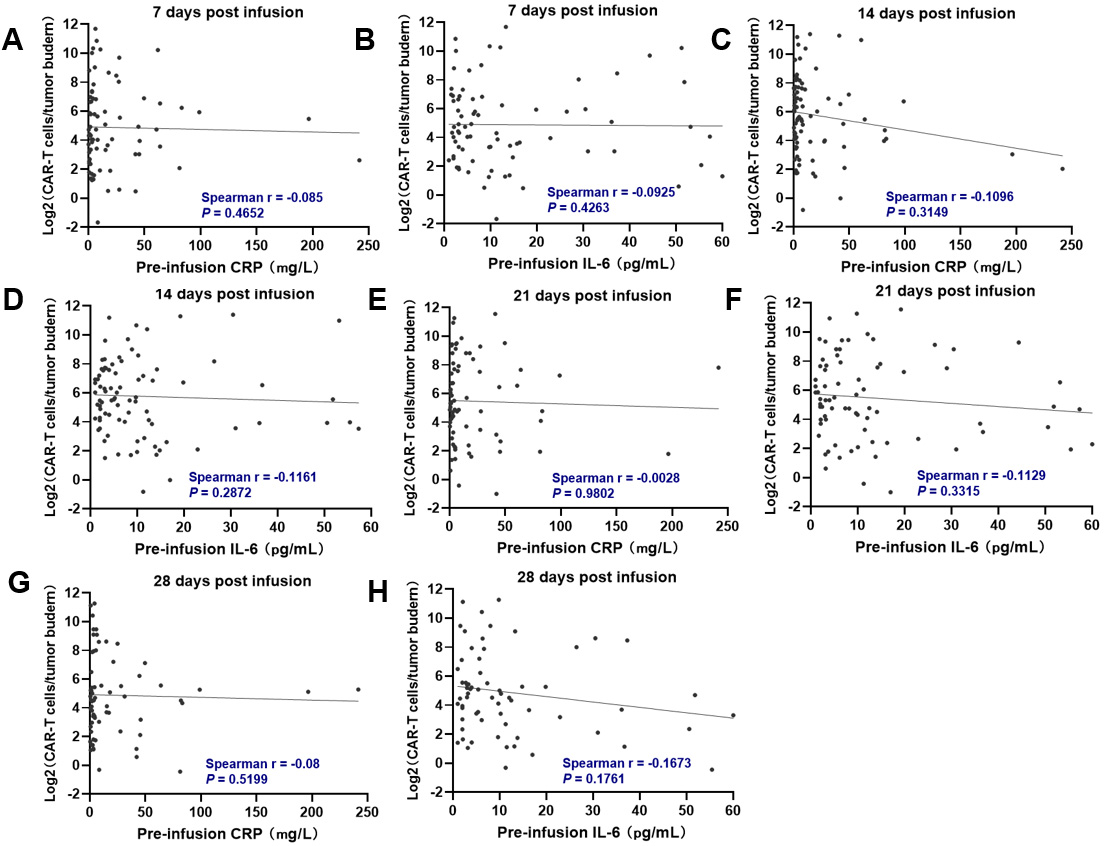
 **Figure S4: Correlation between the pre-infusion inflammatory markers and in vivo CAR T-cell expansion** **normalized to tumor burden during the first month following CAR-T cell infusion.** Tumor burden was defined as percentage of plasma cells in bone marrow. Spearman r value was calculated using the Spearman's correlation test.


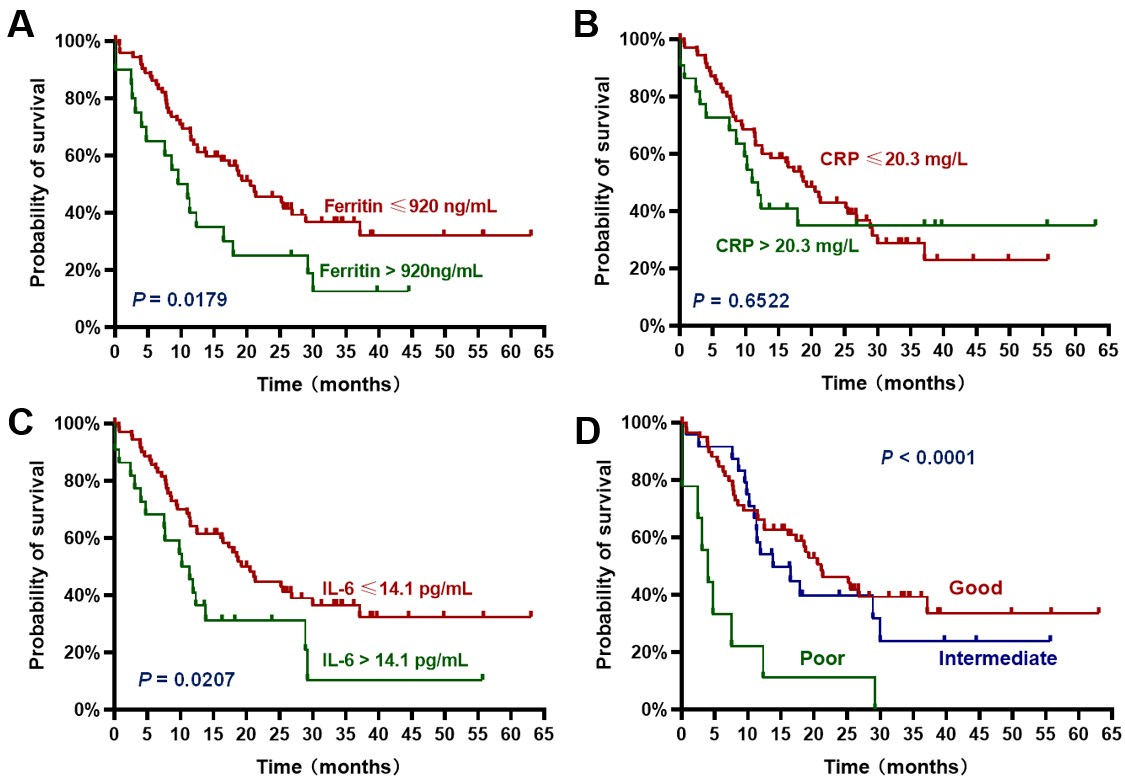


**Figure S5. Duration of response (DOR) according to inflammatory markers and InPI index.** The patients with ferritin > 920 ng/mL (A), IL-6 > 14.1 pg/mL (C) and intermediate to poor InPI (D) had inferior DOR. (B) No significant association was found between CRP and DOR. Survival curves were drawn according to the Kaplan-Meier method. The log-rank test was used to compare the difference in survival probability between two groups.
